# Fatal Case of Perforation of the Duodenum

**Published:** 1843-08-05

**Authors:** Joshua W. Ash

**Affiliations:** Delaware county, Pa.


THE MEDICAL EXAMINER,

And Retrospect of the Medical Sciences.

Vol. VI.] PHILADELPHIA, SATURDAY, AUGUST 5, 1843. [No. 15.

### FATAL CASE OF PERFORATION OF THE DUODENUM.

BY JOSHUA W. ASH, M.D.,

Of Delaware county, Pa.

T. R. æt. 50 years, of strongly marked bilious temperament, energetically active in the prosecution of an extensive business, regular and temperate in his habits, and possessing robust, general health, was attacked suddenly, at 11 o'clock, A. M., June 15th, 1843, while sitting in a meeting for worship, with pain in the epigastric abdominal region of the most intense severity. He had complained for several hours previously of a heavy pain in the right hypochondrium, hard to bear, but not in his estimation sufficiently important to detain him at home. The same had occurred at several different times during the preceding week, which, after lasting a longer or shorter time, had gone off, leaving little or no inconvenience behind. Six weeks previously he had a chill, followed by fever, which confined him but for a day or two. His appetite was good, sleep natural, digestion, assimilation, and other functions healthy; intestinal, urinary, and other secretions perfectly regular, and his whole health, with the above noted exceptions, had been perfectly good up to the moment of attack. I happened to be present, and being called upon, accompanied him home. His pulse was entirely unaffected, his tongue clean and moist, with a skin soft and natural, but which soon became bedewed with copious perspiration, from the intensity of his suffering. His stomach was calm, and he had had a free alvine discharge since breakfast. There existed reducible inguinal hernia of the left side; which was found upon examination entirely free from soreness and pain. In addition to the abdominal pain he complained much of a sense of stricture across the shoulders, extending from before backwards, such as would result from a hard cord drawn tightly across, causing an intensity of suffering, but little less in degree than the abdominal pain. To mitigate the extreme severity of the agonizing pain under which he was suffering, was to my mind the first indication, and one that called imperatively for prompt and decisive action. To effect this object, and with a view at the same time to speedily unload the bowels, I directed full doses of opium, combined with calomel and aided by stimulating enemata. A large sinapism was applied to the abdomen, and blood freely drawn from the arm.

Failing by these measures to afford the desired relief, the injections having in every instance returned without bringing away any fœcal matter, I had him immersed in a warm bath, with only temporary relief. Blood was again drawn freely from the arm, and calomel and opium, in divided doses, alternated with castor oil and sp. terebrinth. directed to be given every two hours until the bowels should be moved, together with fomentations to the abdomen to be steadily and perseveringly applied. Upon taking the third dose of oil his stomach revolted, it was consequently discontinued, and infusion of senna substituted, which, with 2 grs. of calomel, were directed to be continued every two hours, until the desired effect should be produced.

Twelve hours had now elapsed from the time of attack; the pain was mitigated but not subdued; on the contrary, it continued in a degree eminently distressing; the bowels had not yet responded to the measures used for the evacuation of their contents, the epigastric and hypochondriac regions, (particularly the right,) at this time had become in a high degree sensitive to pressure, the sensation produced being, as he expressed it, peculiar and indescribable, but not strictly painful: there being manifest disposition to sleep, I left him for the night, and saw him again at 5 o'clock on the following morning. He had passed much of the night in a state of disturbed sleep, and was still dosing. Not yet was there any improvement in his condition, bowels still unmoved, the pulse had become irritated and accelerated in frequency. Directed a continuance of the treatment, with repetition of the warm bath, I gave a drop of croton oil, to be repeated in an hour should it not have operated, and left him until 9 o'clock, A. M. when I again saw him, and learned there had been a free discharge of blood from the hæmorrhoidal veins accompanying the return of an injection, but without the slightest relief to the symptoms; directed a steady continuance of the treatment till noon, substituting an emollient poultice over the whole abdomen in place of the fomentations, and then, should relief not have been procured, six dozen leeches to be applied to the abdomen. The abdominal sensibility had become increased in acuteness without extending much beyond its former limits; still the sensation produced upon the slightest touch, although unbearable was not that of *pain*, but, in his own words, "a sort of indescribable tickling of the whole insides." His respiration had now a marked character of abruptness. The tongue was still clean and natural, as was also the state of the skin over the whole body. Thirst, which hitherto had not been remarkably urgent, became now incessant, calling for small quantities of liquid. The stomach was still unaffected, with nausea, extreme restlessness, demanded an almost incessant change of posture; the position generally assumed was on the back, slightly inclining to one or the other side, with the head and shoulders elevated to an angle with the horizon of about 30 or 35 degrees.

8 o'clock, P. M. The leeching has not procured for him the slightest relief, but the operation having occupied a long time, has induced a good deal of exhaustion. The bowels still continue obstinately unmoved, and although the pain is not so agonizing as at the beginning, it is still harrasing in no ordinary degree; in short the whole condition of the man is one of unmitigated, indescribable wretchedness. From this time till the hour of his death, which event occurred at 7 o'clock next morning, 44 hours from the time the attack began, was occupied in persevering endeavours to quiet irritation, combat the advancing evidences of inflammation, and procure alvine discharges; but it was all to no purpose, the bowels remaining obstinately closed until the last, not giving issue even to the discharge of gas, until within a very short time of death, when a free discharge of flatus took place. His stomach became sick about four hours before death, from which time he threw up at intervals small quantities of bile-tinged liquid. His mental manifestations were unclouded, and calm throughout the whole period, excepting a few slightly delirious expressions, while in a half sleeping state, when under the influence of opium.

Autopsy, eight hours after death, aided by my friend, Doctor William E. Haines. Present, Drs. Joseph Wilson and A. E. Small.

The exterior of the body presented no marks worthy of particular note. The abdomen, somewhat distended, was more resisting than is usual. Upon cutting into the peritoneal sac there was a rush of gas; its cavity was found to contain a large amount of serous liquid tinged with bile, bearing a close resemblance to the liquid voided from his stomach during the last few hours of life; and, in the more depending parts a heavier matter, of a muco-purulent consistence and appearance. Recently formed imperfect adhesions existed between the abdominal walls and viscera, in the right hypochondriac and contiguous portion of the epigastric region, where the evidences of the highest degree of the inflammatory action were met with. From this point it appeared to have spread as from a centre, and extended itself over the diaphragm, liver, stomach, spleen, and transverse colon—the omentum partaking largely. The whole surface of the liver, spleen and diaphragm, were coated with a layer of purulent matter; the small intestines exhibited but slight traces of inflammatory action. In the progress of the examination it soon became obvious that there existed somewhere a perforation of the intestinal canal, the search for which was commenced at the colic extremity. Passing ligatures around the bowel, it was cautiously separated by the scalpel, and carefully examined throughout its entire length, until, arriving at the duodenum, it was divided and removed. No evidences of disease deserving notice were discovered in any part of the removed portion, consisting of jegunum, ileum, and colon. At this stage of the dissection, happening to notice that the gall-bladder was greatly distended, I passed my finger under its duct, and at the same time making pressure upon its surface, there was an immediate escape of some bilious liquid into the cavity of the belly, which led at once to the discovery of the perforation. This was of a circular form, with clean, even edges, (as though it had been made by a shoemaker's punch,) about as large as a medium sized goose quill. Passing through the coats of the bowel near the entrance of the gall duct, it occupied a position on its concave or left side, about one and a half inches below the pylorus. Passing a ligature around the œsophagus, the stomach and duodenum were removed for closer inspection; laying them open from end to end, the perforation was found to have resulted from ulceration, which, judging from its appearances, was supposed to have occupied a long time in its accomplishment. Through the mucous and muscular coats it was about five-eighths of an inch in diameter, with clean, callous edges, rounded, and much thickened by interstitial deposition—the thickening extending but a little beyond the circumference of the ulcer. The internal coat of the stomach, as well as of the bowels, was apparently perfectly healthy, except only at the seat of ulceration, and for a few lines immediately around it. The gall-bladder was full to repletion, of a very thick, *black*, semi-liquid substance, which appeared to be bile mixed with venous blood. The colon contained a quantity of liquid fæces; and in the small intestines a single lumbricus was found.

---

Dr. Papavoin found that of 532 female children examined at the Hopital des Enfans Malades, Paris, 338, or about two-thirds, had tubercles in the lungs, &c.; while of 387 males, tubercles were found in only 210, or little more than half the number.

*Med. Gaz.*

